# Proceedings of the Central States Dental Association

**Published:** 1866-06

**Authors:** W. H. Shadoan


### Proceedings of Societies.

### PROCEEDINGS OF THE CENTRAL STATES DENTAL ASSOCIATION.

The first semi-annual meeting of the Central States Dental Association, was held in the city of Nashville, Tennessee, on Tuesday, Wednesday and Thursday, April 10, 11 and 12, 1866.

### First Day—Forenoon Session.

The society was called to order by Dr. W. H. Morgan, the President. The minutes of the previous meeting were read; and the roll called, when the following members answered to their names:

Drs. W. H. Morgan, Pres't; W. G. Redman, Vice Pres't; W. H. Shadoan, Sec'y; R. H. Wilson, Treas'r; John Ragland, J. H. Bedford, Jas. L. Nourse, Geo. W. Field, L. E. Wilson, S. J. Cobb, Geo. S. Jones.

Dr. S. J. Cobb being the only member of the Executive Committee present, Drs. R. H. Wilson and J. L. Nourse were appointed to fill vacancies.

The entire Committee of Membership being absent, the President appointed Dr. W. G. Redman, Dr. G. W. Fields, and Dr. L. E. Wilson, to fill said committee.

The Executive Committee then made the following report, which was adopted:

We, the Executive Committee, beg leave to make the following report:

1st. Reading of Essays.

2d. The best means of improving the practice, and elevating the profession of dentistry.

3d. Anæsthetics—their proper use and relative value.

4th. Extracting Teeth — when and under what circumstances should it be done.

5th. Absorption of Alveolar processes—causes and treatment.

6th. Filling Teeth—the relative value of different materials, and the mode of operating in different cases.

7th The best method of obtaining correct impressions, and models of the mouth.

8th. When, and how should the different materials be used for artificial dentures.

9th. Neuralgia—what is it, and how should it be treated?

10th. Miscellaneous Business.

S. J. COBB,
R. H. WILSON,
JAS. L. NOURSE,
*Committee.*

On motion, the hours for meeting shall be from 9 to 1, from 3 to 5, and from 8 to 10 o'clock, each day—adopted.

On motion, adjourned to meet at 3 p.m.

### First Day—Afternoon Session.

President, Dr. Morgan, in the chair. Minutes read and approved.

The Committee on Membership reported the following gentlemen, who were elected members of the Association:

Dr. J. Taft, of Cincinnati, O.; Dr. E. Q. Naghel, of New Albany, Ind.; Dr. R. Russell, Dr. J. C. Ross, Dr. J. M. Buckley, Dr. L. T. Gunn, Dr. E. A. Herman, of Nashville, Tenn.; Dr. A. Hartman, Dr. Thos. Walch, of Murfreesborough, Tenn.; Dr. John Fouche, of Knoxville, Tenn.; Dr. F. W. Garkey, Memphis, Tenn.; Dr. J. T. Noel, Galattin, Tenn.; Dr. C. P. Baird, Winchester, Tenn.; Dr. T. E. Beech, Franklin, Tenn.

The election being over, Dr. Taft offered the following resolution, which was adopted:

*Resolved*, That the Physicians and Surgeons of the city of Nashville, and vicinity, are hereby cordially invited to be present at the sessions of the Central States Dental Association, now being held in this city, at Steadman's Hall, Cherry Street.

A paper from Dr. Gates, of Louisville, Ky., entitled "Dental Education," was presented, and read by the Secretary.

On motion of Dr. Redman, the paper was received and filed.

The subject of the paper being the first subject for discussion, it elicited considerable remarks from nearly all present; and all seemed to deeply feel the importance of a thorough dental training, both as a protection to dentist and patient.

On motion, adjourned to 7½ o'clock, p.m.

### Evening Session.

Minutes read and approved.

The subject of dental education was then taken up and further discussed.

During the remarks on dental education, Dr. Taft made mention, that the people and dentists of Ohio had petitioned the Ohio Legislature to pass a law making it a misdemeanor for any one to practice dentistry, in that State, unless he be a graduate of some Dental College, or undergo a satisfactory examination by a board of dentists, appointed by the Governor for that purpose, or having had a successful practice for ten years or over.

(The Doctor did not say whether or not, the law to be passed, required them to practice the ten years in the State of Ohio,—*Sec'y.*) The Doctor was very confident it would pass, at the next session of the Legislature, and become a law.

The idea of Ohio passing such a law, created quite a stir among the Kentucky and Tennessee dentists, as they were not friendly to the sending of all the *Quacks* from Ohio to these States. They felt that they had enough now; and to prevent others from coming, as well as to get rid of some of those they now have, they determined to set about the work of having a similar law passed in their States.

On motion, the next subject, Anæsthetics, was taken up and discussed at some length.

All concurred, that it was best to use as little chloroform as possible.

Adjourned to meet at 9 a m., to-morrow.

SECOND DAY—FORENOON SESSION.

Minutes read and approved.

On motion of Dr. R. H. Wilson, that the minutes be so altered as to read, that chloroform should be used with great caution—carried.

On motion, the 4th subject " Extracting Teeth," was then taken up, and discussed.

On motion, the next subject was then taken up, "Absorption and reproduction of Alveolar process," and discussed.

Some of the members were of opinion, that the reproduction of bone was a matter in theory rather than practice, and seemed to think that no such thing could be done.

Dr. Taft occupied the time for some minutes, in showing how such a thing was done, which he did in a very clear and forcible manner. He showed that the reproduction of alveolar process, was not only possible, but practicable, and that to his own knowledge, it had been done, not only once, but divers times.

Dr. R. H. Wilson, made some explanation of his views of what was contemplated in the subject. That when any part of the alveolar process was entirely gone, that it could not be restored. But when the destruction was only partial, that it might be done.

Here, Dr. S. J. Cobb introduced Drs. Meenes, Beaty, and Du Pree. On motion of Dr. Cobb, these gentlemen were invited to take part in the discussions—carried.

The President announced the following essayists for the next meeting:

Dr. E. Q. Naghel—Importance of preserving children's teeth.

Dr. Fouche—Creosote, its value, and uses in dentistry.

Dr. Baird, of Winchester, Tenn.—Duties of patients and dentists to each other.

Dr. Garkey—Improvements in Dentistry.

Dr. Shadoan—How to obtain an appreciative and remunerative practice.

Dr. Nourse—Alveolar Abscess.

Dr. Redman—Specialties in Dentistry.

On motion, when we adjourn, we visit the tomb of Ex-President Polk, in a body.

Adjourned to meet at 3 o'clock, p. m.

SECOND DAY—AFTERNOON SESSION.

Minutes read and approved.

The subject of absorption was again taken up, and on motion, it was passed by for the present, to be taken up at some future time, and the 6th subject was taken up—nearly all present taking part.

Dr. Naghel was of opinion that of all cheap materials, amalgams and tin, were the best. He has two fillings in his teeth, (one tin and one amalgam), that were put there when he was only ten years old, in 1837.

On, motion, the Physicians of the city be requested to meet with us this evening, and take part in discussing the subject of absorption and reproduction—carried.

Adjourned to meet at 8 o'clock, p. m.

### SECOND DAY—EVENING SESSION.

Minutes read and approved.

The subject ot absorption and reproduction being again taken up, Dr. Taft spoke at some length. His remarks were of deep interest, and produced a fine effect. He recommends phosphate of lime in difficult dentition, as well as in the reproduction of processes.

The subject being pretty well ventillated, it was discontinued, and the next subject, filling teeth, resumed.

Dr. Garkey—In case of destroying the pulps of the teeth, does not carry it to the same extent he once did. He applies arsenious acid for a few hours, then removes pulp to fangs of tooth, and leaves the nerve in fangs. If they bleed freely, so much the better. Applies a little collodion on cotton, and places it on the bottom of cavity, and lets it remain for three or four days, when he generally finds the membrane nicely healed over; then plugs as usual.

Dr. Shadoan—thinks Dr. Garkey's practice, in such case, good. Where a tooth, say a second superior molar, is decayed on the posterior lingual surface, near the gum exposing the nerve, he drills a hole into the pulp through the centre of the masticating surface of the tooth, and enlarges freely. Then he finds little or no trouble in thoroughly removing every particle of nerve membrane. Would rather fill that cavity made by the drill for nothing, than to undertake to make a successful operation from the cavity of decay. Thinks no material fit to fill teeth with but gold; but if cheap materials have to be, or will be used, prefers Wood's fusible alloy, and tin, to all others. Does not use amalgams at all.

The following resolution was then offered and adopted, by Dr. G. W. Fields:

*Resolved*, That the wives of the dentists of this Association, and of the physicians of the city, or any other ladies who may wish to attend, are respectfully invited to be present the forenoon session of this Association on to-morrow.

The Association then adjourned to 9 o'clock, a. m., to-morrow.

THIRD DAY—FORENOON SESSION.

Minutes read and approved.

On motion, the 6th subject was passed and the 7th taken up, "Impressions of the mouth, &c." Nearly all present described their manner of taking impressions, which was quite interesting. A majority use plaster of Paris.

On motion, the 8th subject, "Artificial Dentures," was taken up. Nearly all present taking part in the discussions.

On motion, the 9th subject, "Neuralgia," was discussed. Dr. Taft described Neuralgia, and gave the treatment.

On motion, adjourned to meet at 2 p. m.

THIRD DAY—AFTERNOON SESSION.

Minutes read and approved.

On motion, miscellaneous subjects were taken up, and after several subjects, not in the regular order, were glanced over, the discussions closed.

On motion, the following resolution, offered by Dr. J. L. Nourse, was adopted:

*Resolved*, That the Executive Committee be requested to report through the Secretary, the subjects for discussion at our next meeting, at least two months previous to said meeting.

Dr. W. H. Morgan proposed to give a reward of fifty ($50) dollars, for the most approved essay on "How to promote the health of the dentist,"—subject to same rules and regulations as the prize essays offered by the Mississippi Valley Dental Association.

The committee appointed to decide upon the merits of the essay, was appointed, and is composed of the following gentlemen: Drs. Driggs, Rogers, and Taft.

On motion, the following resolution, offered by Dr. R. H. Wilson, was adopted:

*Resolved*, That the thanks of this Association are hereby tendered Mr. Steadmen, for the use of his rooms during the sittings of this Association.

On motion, the following resolution, offered by Dr. Redman, was adopted:

*Resolved*, That the thanks of all visiting dentists are hereby tendered to Dr. Morgan, our President, and the dentists of Nashville, for the very kind manner in which they have received and treated them during their stay. And all the members of the Association, to Dr. Morgan, the President, for the able manner in which he has presided over this society.

On motion, the Association adjourned, to meet in the city of Louisville, on the second Tuesday in July, next, it being the 17th day, 1866.

W. H. SHADOAN, *Sec'y.*

